# Frequencies or Absolute Numbers? Cluster Analysis of Frequencies and Absolute Numbers of B-Cell Subsets in Dialysis Patients Who Are Candidates for Kidney Transplantation Reveals Different Profiles

**DOI:** 10.3390/jcm13216454

**Published:** 2024-10-28

**Authors:** Ariadni Fouza, Asimina Fylaktou, Anneta Tagkouta, Maria Daoudaki, Lampros Vagiotas, Efstratios Kasimatis, Aliki Xochelli, Vasilki Nikolaidou, Georgios Katsanos, Georgios Tsoulfas, Lemonia Skoura, Aikaterini Papagianni, Nikolaos Antoniadis

**Affiliations:** 1 Department of Transplant Surgery, Center for Research and Innovation in Solid Organ Transplantation School of Medicine, Aristotle University of Thessaloniki, 54124 Thessaloniki, Greece; ariadnefou@gmail.com (A.F.); lampisv@yahoo.gr (L.V.); katsanosg@auth.gr (G.K.); tsoulfasg@auth.gr (G.T.); nikanton@auth.gr (N.A.); 2 Department of Immunology, National Peripheral Histocompatibility Center, Hippokration General Hospital of Thessaloniki, 54642 Thessaloniki, Greecealiki.xochelli@gmail.com (A.X.); basoniko@hotmail.com (V.N.); 3 Laboratory of Biological Chemistry, School of Medicine, Aristotle University of Thessaloniki, University Campus, 54124 Thessaloniki, Greece; taganneta@hotmail.com; 4 1st Department of Nephrology, School of Medicine, Aristotle University of Thessaloniki, 54124 Thessaloniki, Greece; frasci@outlook.com.gr (E.K.); aapapagi@auth.gr (A.P.); 5 Department of Microbiology, AHEPA Hospital, School of Medicine, Aristotle University of Thessaloniki, 54124 Thessaloniki, Greece; lemskour@auth.gr

**Keywords:** cluster analysis, dialysis patients, double-negative memory B cells, memory Bregs, transitional Bregs, dialysis vintage

## Abstract

**Background:** Detailed characterization of B cells in dialysis patients who are candidates for kidney transplant is still lacking, with little information on how dialysis duration and modality impact B cell subsets. **Methods:** Cluster analysis of flow cytometry determined the frequencies and absolute numbers of B-cell subsets and divided the cohort of 78 candidates into two distinct clusters, one with shorter and one with longer dialysis duration. **Results:** The immune profiles of the clusters differed depending on whether frequencies or absolute counts were considered. In long-term dialysis patients, the frequency of total memory, double negative and marginal zone B cells increased, while the frequency of naive and regulatory B cells decreased. This pattern was reversed in short-term dialysis patients, with a decrease in memory and an increase in naive and regulatory populations. The B subset number decreased significantly in long-term dialysis patients, while it increased significantly in short-term dialysis patients. The dialysis modality affected the frequency-based subset immune profiles. **Conclusions:** It is important to determine whether the evaluation is based on frequencies or absolute numbers. The different distribution of B cell subsets in the clusters, in terms of frequencies and absolute numbers, was influenced by dialysis duration. Modality and age only influenced the frequencies.

## 1. Introduction

Kidney transplantation represents the optimal treatment for patients with end-stage renal disease (ESRD), offering the most favourable long-term survival and quality of life outcomes [1]. However, due to the limited availability of suitable kidney grafts, the majority of ESRD patients are required to undergo extended periods of dialysis therapy and lengthy waiting periods for kidney transplantation. The ESRD patient population is heterogeneous, comprising individuals with diverse aetiologies of renal failure. However, they are unified by impaired immune system function. They exhibited uraemia-related immune system dysfunction [2,3,4], which is further exacerbated by dialysis, with the potential for adverse effects on long-term transplantation outcomes [5,6,7].

The majority of studies examining immune system dysfunction in these patients have focused on the impairment of T cell function [5,8,9,10]. There is currently a lack of information available regarding the comprehensive characterisation of circulating B cells and their subsets in dialysis patients who are candidates for kidney transplantation. Our objective was to determine the frequencies and absolute numbers of B-cell subsets in those patients, as well as the impact of dialysis and dialysis vintage and the influence of dialysis modality on those. To this purpose, a multiparametric flow cytometric approach was employed.

We selected a control group of pre-emptive kidney transplant candidates from our cohort. Pre-emptive candidates showed functional and phenotypic changes in peripheral lymphocytes [5,9]. This approach was chosen over the use of healthy individuals because healthy individuals are unable to adequately reproduce the immune system perturbations caused by the uraemic environment [3,4,11]. 

Given that the analysis of immune cells in various studies is predominantly based on percentage data, with both percentage and absolute count data used to a lesser extent [12,13,14], the aim of our study was to use hierarchical cluster analysis to categorize the cohort of dialysis patients candidates for kidney transplantation into distinct groups based on the frequency and absolute number of immunophenotypes of B-cell subsets. This enabled the identification of specific patient immune profiles. These profiles were then compared with those of pre-emptive candidates to determine the impact of haemodialysis on B cell phenotypes. Furthermore, this study correlated these profiles with varying periods of dialysis, age of the patients, and modality of haemodialysis.

Two distinct clusters were identified in each case. The composition of the patients and their immune profiles differed in the different clusters, as did the dialysis vintages and the age of the patients. The identified immune profiles were compared with those of the pre-emptive candidates to determine the impact of haemodialysis on the immunophenotypic profile of B-cells.

## 2. Methods

### 2.1. Laboratory Studies

The testing included the determination of serum creatinine levels, leukocyte and lymphocyte counts, which were used to determine the absolute counts of the subsets under investigation.

### 2.2. Study Population

A total of 85 patients were enrolled in this prospective, observational single-centre cohort study. The majority, 78, were undergoing dialysis, while 7 had been pre-emptively enrolled (without receiving dialysis) for kidney transplantation. As part of the enrolment process, clinical data and blood samples were obtained from each patient.

The dialysis vintage was defined as the period between the date of haemodialysis initiation and the date of transplant surgery.

#### 2.2.1. Inclusion Criteria

Eligible participants were adults between the ages of 18 and 60 years on dialysis treatment or without dialysis treatment kidney transplant candidates, all of whom had been under regular follow-up in the Transplant outpatient clinic.

#### 2.2.2. Exclusion Criteria

Patients were excluded from the study if they had a history of malignancy, autoimmune disease, haematological disease, or had undergone treatment with monoclonal antibodies against B or T lymphocytes for a period of less than five years. Additionally, those who had been diagnosed with cytomegalovirus, Epstein-Barr virus, or other viral pathogens or bacterial infections within the previous three months were also excluded.

### 2.3. Study Schedule

**Figure 1:**
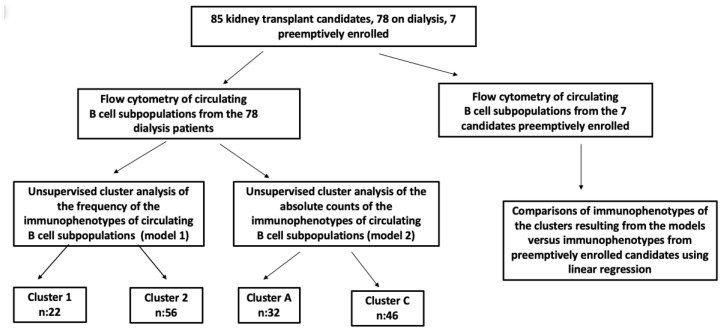
Workflow of the study.

Each candidate was included in the study according to the above-mentioned inclusion criteria. The demographic and clinical data (Table 1), as well as the data pertaining to the medical history and primary disease, were obtained from the patient records. The workflow diagram is shown in Figure 1.

### 2.4. Ethics Approval and Consent to Participate

This study was conducted in accordance with the ethical regulations set by the Declaration of Helsinki and with the approval of the Institutional Review Board of the Medical School at Aristotle University of Thessaloniki (reference number 4356, approval date 26 January 2021). Each patient gave their written informed consent.

### 2.5. Flow Cytometry Method to Profile Circulating B Cell Phenotypes

Phenotyping of B cells and their subsets, naïve B cells, memory cells: class-switched class-non switched, double negative, the peripheral equivalent of marginal zone B cells and B cells with regulatory properties including plasmablasts, transitional Bregs and memory Bregs, was conducted using flow cytometry on peripheral blood samples [15]. The samples were subjected to the previously described staining procedure [16,17] with fluorochrome-labelled monoclonal antibodies, Supplement Table S1. Subsequent analysis of the samples was conducted on an 8-colour Navios flow cytometer (Beckman Coulter, Marseille, France) and data processing was performed with the Kaluza program (Beckman Coulter, Marseille, France).

The IgD/CD27 classification system [18] was employed to distinguish between naïve B cells (CD19+IgD+CD27−) and memory B cell subsets, including total memory B cells (CD19+CD27+) and their subsets: class-switched memory B cells (CD19+IgD−CD27+) and class non-switched antigen memory B cells (CD19+IgD+CD27+), Table S1. B cells with memory properties (CD19+IgD−CD27−) were identified based on the loss of both surface markers, IgD and CD27. To gain further insight into the distribution of circulating B cell subsets, staining was performed using the combined expression of the surface markers CD38, CD24 and IgM. The differential expression of markers CD19, CD24, CD27, CD38, IgD, and IgM enabled the identification of the peripheral equivalent of marginal zone B cells (CD19+CD27+IgD+IgM+) and B cells with regulatory properties, including the identification of plasmablasts (CD27++CD38++IgD+/−), transitional Breg (CD24++CD38++) and memory Breg cells (CD24++CD27+). The frequency of these subsets within the total CD19+ B cell population was also determined.

### 2.6. Cluster Analysis

A cluster analysis was conducted on the frequencies and absolute counts of B-cell subsets in 78 dialysis patients who were candidates for transplantation.

**Figure 2:**
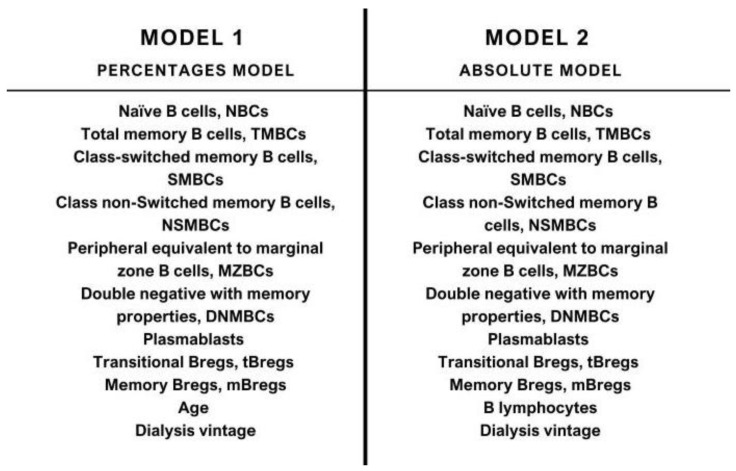
The two models constructed by cluster analysis.

The Agglomerative Hierarchical Clustering (AHC) algorithm, which involves the continuous merging of data vectors (in this case, transplant candidates) in accordance with predefined distance criteria, was employed to analyse the data. The initial model focused on frequency (Model 1) and the subsequent model examined the absolute counts (Model 2) of the B cell subsets (considered as continuous variables). In addition, the models included transplant candidates’ dialysis vintage and age. Additional information on the cluster analysis is included in the Supplementary Materials (with the addition of Figure 2, removed from the results).

### 2.7. Statistics

The statistical distribution of the quantitative variables was assessed using the Shapiro–Wilk normality test and graphical inspection of the variables. Levene’s test was employed to test for equality of variances.

The regression assumptions of the linear regression models used in all cases were tested, and if they were not met, the model was either modified or rejected. A Kruskal–Wallis test (non-normal distribution) was employed for comparisons between more than two unrelated groups, while Dunn’s post hoc test used following group comparisons. Categorical variables were compared using Fisher’s exact test for contingency tables. Values with a P less than 0.05 considered significant in all cases. The Bonferroni correction was used for multiple comparisons.

All statistical comparisons were performed in RStudio 2023.12.0.

## 3. Results

### 3.1. Two Different Models Were Identified Using Clustering Analysis

The final variables in each of the two clustering models are presented in Figure 2 and are discussed in subsequent sections of this article.

### 3.2. Cluster Analysis Based on the Frequencies (Percentages, Model 1) of B Cell Subsets Identified Distinct Groups of Dialysis Patients Who Were Candidates for Kidney Transplantation

**Figure 3:**
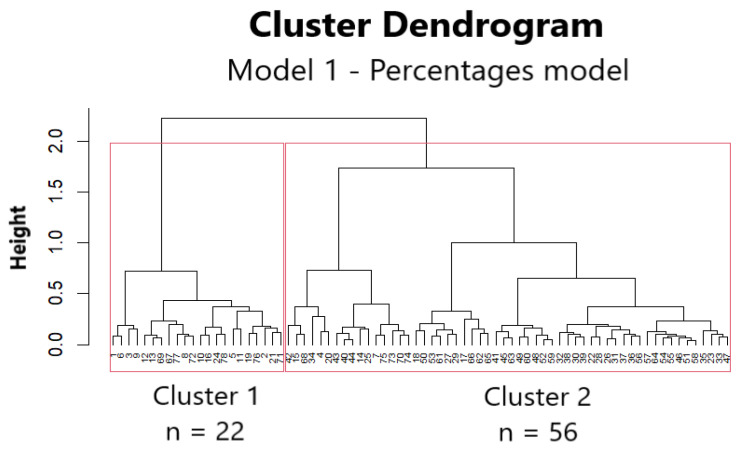
Cluster dendrogram of Model 1 (percentage model). The two clusters are shown as rectangles, separating 22 patients (cluster 1) from 56 patients (cluster 2). The vertical axis of the dendrogram (height) represents the dissimilarity between the clusters, as expressed by the distance metric. The greater the vertical distance, the greater the dissimilarity. The horizontal axis represents the individual data points (patients) that are grouped into clusters.

A cluster analysis of frequencies classified the 78 kidney transplant candidates into two discrete patient groups, designated as clusters 1 and 2, Figure 3.

The characteristics of clusters 1 and 2 are shown in Table 2.

**Figure 4:**
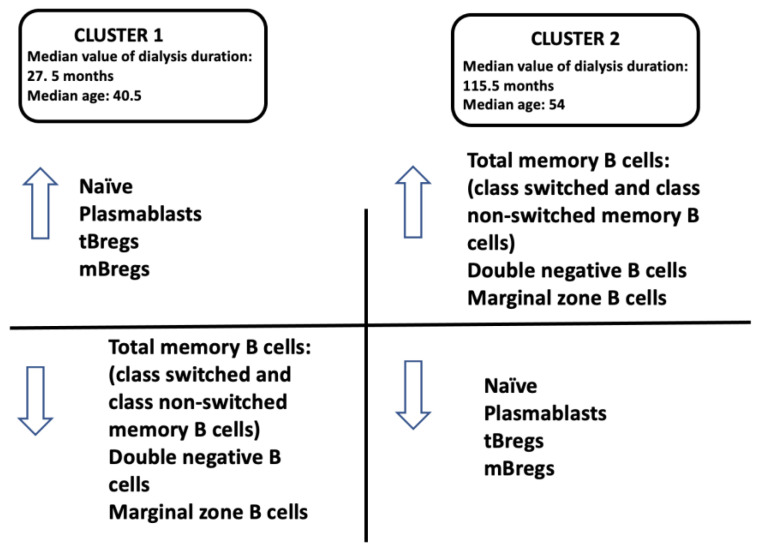
The cell immune profile of candidates in cluster 1 and 2 (Model 1).

Twenty-two (22) candidates comprised cluster 1, representing 28% of the total sample, with a median dialysis vintage of 27.5 months, a median serum creatinine level of 5.1 mg/dL, and a median age of 40.5 years. Of these candidates, 15 (68%) were on standard haemodialysis and the remaining 32% were on CAPD. The immune profile of cluster 1 transplant candidates was characterized by a high frequency of naive, plasmablasts, tBregs and mBregs and a low frequency of total memory B cells, switched and non-switched memory B cells, double negative cells and marginal zone B cells (Figure 4).

Cluster 2 included 56 candidates (72% of the total sample), with a median dialysis vintage of 115.5 months, a median serum creatinine level of 4.9 mg/dL, and a median age of 54 years. Of these candidates, 91% were on classical haemodialysis, while the remaining 9% were on CAPD. The immune profile of cluster 2 transplant candidates was characterized by an increased frequency of total memory B cells, switched, and non-switched memory, double negative cells and marginal zone B cells, and a reduced frequency of naive, plasmablasts, tBregs, and mBregs (Figure 4).

Therefore, the frequency distributions of the B-cell subsets differed between clusters 1 and 2 and dialysis duration had a significant effect on the B-cell subset distribution as a variable of the clustering model, which differed between the two clusters according to the model evaluation with linear regression. The linear regression models used to assess the model and validate its results are presented in Table S1 (Supplement).

To determine whether it is the frequencies or the absolute numbers that should be considered for evaluation, the absolute numbers of B lymphocyte subsets in patients belonging to clusters 1 and 2 (Model 1) were compared. The identified differences were validated, resulting in the production of different immune profiles in terms of frequency and absolute numbers of B cell subsets from the same population.

As shown in Table 3, the analysis revealed a statistically significant increase in the absolute number of memory cell counts in patients on long-term dialysis compared to those on short-term, with a similar trend observed in terms of frequency. The absolute numbers of naïve cells were comparable between the two clusters, but the frequency was significantly lower in patients on long-term dialysis than in the other group. There was a notable increase in MZB counts in patients on long-term dialysis compared to those on short-term, with no significant difference in frequency between the two groups. While there was no significant difference in the frequency and numbers of mBregs, there was a notable decline in the absolute number of tBregs in patients on long-term dialysis compared to those on short-term dialysis. There was no significant difference in terms of frequency.

It is therefore important to consider both the frequency and the absolute number of B cell-associated immune profiles generated from the same population when interpreting the data, as the results may be different depending on the approach taken.

### 3.3. Comparison of Frequencies of B Cell Subsets in Dialysis Patients, Kidney Transplant Candidates and Pre-Emptive Candidates

There were frequency differences in B-cell subsets between the dialysis groups and pre-emptives. While these differences did not reach statistical significance overall, they were statistically significant for plasmablasts (median, IQR) for dialysis patients: 4.1 (1.5, 6.15) and for pre-emptive patients: 0 (0, 0.97), Table 4.

It is acknowledged that the sample size of the pre-emptive candidates is small, which introduces limitations to the study.

### 3.4. Comparison Between Clusters 1 and 2 and Cluster of Pre-Emptives

We constructed linear regression models for comparing cluster 1, cluster 2 and the pre-emptive patients (Supplementary Materials). A notable difference was observed in the composition of clusters 1 and 2 and the pre-emptive patients, as evidenced by linear regression with a *p*-value less than 0.004 (*p* < 0.004, Bonferroni correction).

Cluster 1 was distinguished by a younger age profile, with a median age of 40.5 years (Table 2), significantly lower than the median age of 54 years in cluster 2 (*p* < 0.001, Table S2). No significant difference in age observed when pre-emptive patients were compared to the model clusters (Supplement Table S3).

A significant difference was observed in dialysis vintage (months of dialysis) between cluster 1 and 2 (Table 2), with cluster 1 having a lower dialysis vintage (*p* < 0.001, Table of linear regression, Table S2).

### 3.5. Cluster 1 vs. Cluster 2

Short-term dialysis candidates (cluster 1) showed increased frequencies of naive, plasmablast, tBregs, mBregs and decreased frequencies of total memory B cells, class switched memory B cells, class non-switched memory B cells, double negative B cells and marginal zone B cells. In cluster 2 (long-term dialysis candidates) the frequency pattern was reversed, with a high frequency of total memory B cells, class switched memory B cells, class non-switched memory B cells, double negative B cells and marginal zone B cells and a low frequency of naive, plasmablasts, tBregs, and mBregs.

A statistically significant difference was observed in the naïve, total memory, switched, non-switched memory, plasmablast and tBreg populations. In contrast, the remaining populations of interest, namely DNs, MZBs, and mBregs, demonstrated no significant difference (Table 2).

### 3.6. Cluster 1 vs. Pre-Emptive

No significant differences were observed in the subpopulation variables when cluster 1 and the pre-emptive candidates were compared.

### 3.7. Cluster 2 vs. Pre-Emptive

The frequency of plasmablasts and tBregs differed significantly between cluster 2 and pre-emptive patients, while no significant differences were observed in the remaining subsets.

The results of this study indicated that the immune profile of B cell subset frequencies in dialysis patients was comparable to that of pre-emptive candidates, with the exception of those of plasmablasts and tBregs, which exhibit a statistically significant difference (*p* < 0.001; *p*-value = 0.004 after Bonferroni correction, Table S3) between pre-emptive candidates and those of long-term dialysis patients. The tBregs frequency was higher in the pre-emptive transplant candidates (Table S3).

### 3.8. Comparison Between Variables That Are Not Included in Model 1

#### 3.8.1. Creatinine Levels

The creatinine levels in clusters 1 and 2 were comparable, with no significant difference (5.1 vs. 4.9 mg/dL in cluster 2). Pre-emptive patients also showed no significant difference when compared with the Model 1 clusters (Table S3).

#### 3.8.2. Dialysis Modality

In terms of dialysis modality, cluster 1 comprised 15 subjects (68%) on standard haemodialysis, while the remaining 32% were on CAPD. Cluster 2, by contrast, comprised 91% (51 candidates) on haemodialysis and the remaining 9% (5 candidates) were on CAPD. Cluster 1 had a higher proportion of CAPD patients compared to cluster 2, which had a higher proportion of haemodialysis patients than cluster 1. The two clusters were found to differ significantly in the modality of dialysis (*p* < 0.05), as shown in Table 2. This clearly demonstrated that there was a significant difference in how patients were distributed between HD and CAPD across the two clusters.

### 3.9. Cluster Analysis Based on the Absolute Numbers (Model 2) of B Cell Subsets Identified Distinct Groups of Dialysis Patients Who Were Candidates for Kidney Transplantation

**Figure 5:**
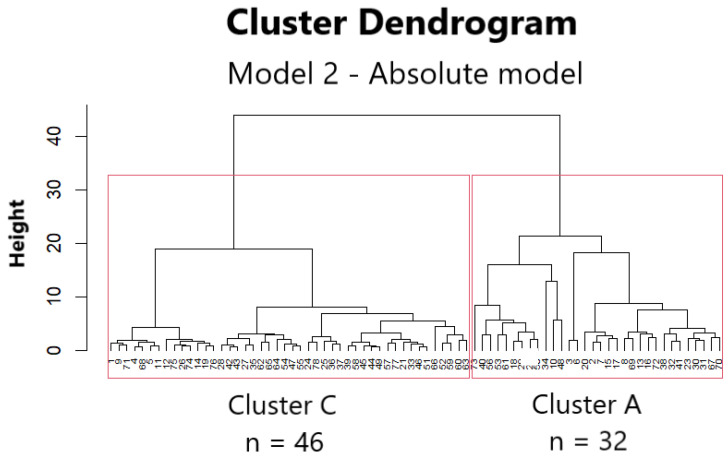
Cluster dendrogram of Model 2 (absolute model). The two clusters are shown in rectangles separating 46 patients from 32 patients (left; cluster C, right; cluster A). The vertical axis of the dendrogram (height) represents the dissimilarity between the clusters as expressed by the distance metric. The greater the vertical distance, the greater the dissimilarity. The horizontal axis represents the individual data points (patients), which were combined to form clusters.

The analysis on the basis of B cell absolute numbers from 78 candidates yielded two discrete clusters A and C (Figure 5), with the distribution of B-cell subsets in clusters A and C shown in Table 5.

#### 3.9.1. Comparison Between Cluster A, Cluster C and Pre-Emptives

##### Cluster A vs. Cluster C

Cluster C comprised 46 individuals (representing 59% of the sample), with a median duration of dialysis of 112.5 months, statistically higher (*p* = 0.002) than that of the 62.5 months observed in cluster A, which included 32 individuals (41% of the sample).

**Figure 6:**
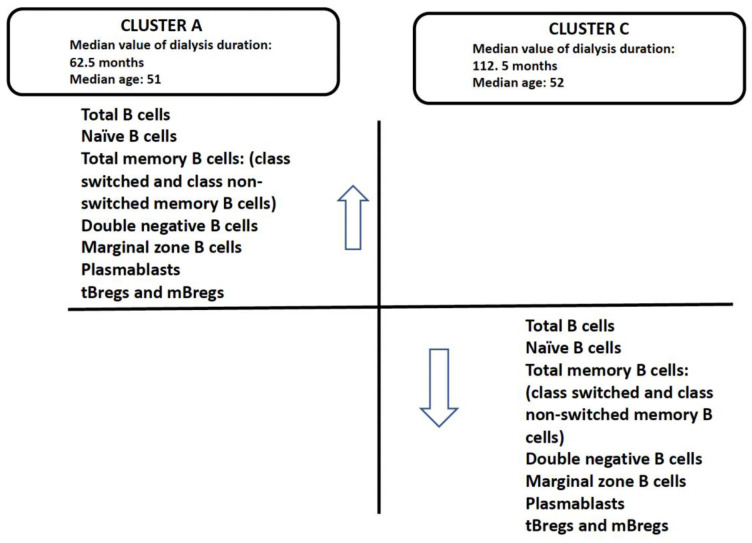
The cell immune profile of candidates in cluster A and C.

The profile of the B cell subsets demonstrated an inverse relationship between the absolute number of cluster A and the profile derived from the cluster C data (Figure 6). A significant decline in the number of B cells in cluster C was observed in comparison to cluster A, attributed to a general reduction across all subsets. The profile of cluster A was characterised by an increased number of B cells and their subsets.

These findings suggested a potential relationship between dialysis duration and absolute count levels, with shorter dialysis periods being linked to higher absolute count levels.

##### Cluster A, Cluster C vs. Pre-Emptives

Seven candidates were identified as pre-emptive. Significant differences were observed in the absolute numbers of different subsets between clusters A and C, as well as pre-emptive patients (*p* < 0.004, Bonferroni correction, Table S4).

##### Cluster C vs. Pre-Emptives

A statistically significant difference was observed in the case of plasmablasts between the pre-emptive patient group and those belonging to cluster C. However, no such difference was evident in the case of the remaining subsets.

##### Cluster A vs. Pre-Emptives

The data revealed a significant difference between the levels of plasmablasts and mBregs observed in Cluster A and those observed in the pre-emptive group. However, no significant differences were noted in the remaining subsets.

The results of this study demonstrated that the immune profile of the B cell subset absolute numbers in dialysis patients was comparable to that of pre-emptive candidates, with the exception of plasmablasts for all dialysis patients and mBregs for short-term dialysis patients.

### 3.10. Comparison Between Variables That Were Not Included in Model 2

#### 3.10.1. Creatinine Level

The creatinine levels in cluster A, C and cluster of pre-emptives showed no statistically significant difference (4.7, 5.1, and 4.3 mg/dL) (Table 5).

#### 3.10.2. Modality of Dialysis

With regard to the modality of dialysis, cluster C comprised 39 subjects (85%) on standard haemodialysis, while the remaining 7 (15%) were on CAPD. Cluster A comprised 27 candidates (84%) on classical haemodialysis, with the remaining 16% (5 candidates) on CAPD. The two clusters did not exhibit a statistically significant difference in the modality of dialysis (Table 5). The B cell subsets studied showed no difference between different groups of dialysis treatment.

#### 3.10.3. Age

In terms of the age of patients between the two clusters of Model 2, the results did not show a significant difference between them.

## 4. Discussion

Patients who have reached a stage where dialysis is a necessary treatment for a variety of underlying causes represent a highly diverse cohort with a number of distinct changes in their immune systems. There is currently limited literature on the topic of B cell immunophenotypes in dialysis patients undergoing evaluation for kidney transplantation, with a particular focus on the duration of their dialysis treatment and dialysis modality.

In this study, cluster analysis was employed to categorize dialysis patients who were kidney transplant candidates into distinct groups based on the frequency and absolute numbers of the immunophenotypes of B-cell subsets. This was performed with the aim of identifying B-cell-associated immune profiles among patients with different dialysis durations and types of dialysis.

The results demonstrated that there were quantitative changes between cellular populations in the clusters produced in terms of both frequency and number. It was determined that dialysis duration, and the modality, had an impact on B cell subsets, with varying degrees of effect.

The frequency of naive cells showed a significant difference between the two dialysis groups, with duration of dialysis having opposite effects on the frequency, increasing in patients with a shorter duration of dialysis and decreasing in patients with a longer duration of dialysis. No difference was observed between pre-emptive and dialysis patients.

B cell memory frequency showed a significant increase in long-term dialysis patients compared to short-term patients, probably due to their prolonged exposure to a significant number of pathogens, resulting in the development of increased cellular memory. No statistically significant difference was found between dialysis and pre-emptive candidates, but the latter had a higher frequency of memory cells compared to short-term patients. This higher frequency was attributed to non-specific activation of the immune system as a complication of uraemic conditions. However, with increasing duration of dialysis, the frequency of memory cells increased and reached a level comparable to that observed in pre-emptive patients. Our results differed from those of Kim et al. [19], who reported a significant increase in memory cells in patients not on dialysis compared to those on dialysis. The discrepancy between the two studies may be due to a number of factors, including the relatively small sample size of pre-emptive patients in our study.

Furthermore, in the two groups of patients with different durations of dialysis, the frequency of MZBs differed, but did not reach significance, with higher frequencies observed in long-term dialysis patients. These results, consistent with the findings of Stranavova L. et al. [11], were expected, as MZB cells act as a first line of defence against encapsulated bacteria and the removal of altered self-antigens [20,21]. No significant difference in the frequency of pre-emptive candidates was observed compared to both groups of dialysis patients.

For tBregs, there was a significant difference between the two dialysis groups, with a lower frequency in the long-term patients. The observed significantly higher frequency in pre-emptive patients compared to long-term dialysis patients probably contributes to the homeostasis of the immune system under uraemic conditions. Patients with a shorter dialysis duration and pre-emptive patients had comparable frequencies of tBregs, suggesting that tBregs were less affected by dialysis. This was consistent with the results reported by Pahl MV et al. [22] but not with those of im et al. [19] who found a significant reduction in tBregs in non-dialysis patients compared to dialysis patients. In contrast, Stranavova et al. [11] found no significant difference between pre-emptive and dialysis patients over a dialysis period of more than three months. These discrepant results could be explained by the differing cohorts studied.

With regard to the regulatory population of mBregs and dialysis, there are currently no published data available. Our results indicated that there was no difference between the dialysis groups. The clinical significance of this population is currently under investigation.

Data on plasmablasts showed a significant higher frequency in pre-emptive patients compared to longer-term dialysis patients. This higher frequency was probably due to uraemic toxicity, which activates the immune system.

Dialysis and its duration did not affect the frequency of double-negative B cells.

The duration of dialysis treatment had a contrasting effect on the absolute number of B-cell subsets. Patients on short-term dialysis showed a significant increase in the absolute number of B-cell subsets, whereas patients on long-term dialysis showed a decrease.

To improve the interpretation of the data, we performed an evaluation of the immune profile derived from the absolute numbers of B cell subsets within the two clusters formed on the basis of frequencies. This resulted in the generation of an immune profile with altered statistical discrimination of differences between subsets compared to the frequency profile. The results indicated that it is important to consider both the frequencies and the absolute numbers of immune phenotypes of the study populations when evaluating cases.

Given the inherent variability in immune cell frequencies and numbers with age [23], and despite conflicting findings regarding B lymphocytes [9], we performed a regression model analysis to assess the impact of patient age on B cell subsets. Our results showed that patient age contributed to the observed changes in frequencies, whereas the absolute numbers of the B cell subsets in the short-term dialysis group (cluster A) and long-term dialysis group (cluster C) were not affected by patient age. This finding was in agreement with Chen, X. et al. [10], who suggested that age-related changes in immune function in dialysis patients were predominantly seen in T-cell population counts, with a less pronounced effect on B-cells.

Data from a limited number of individuals on peritoneal dialysis in this study showed that the dialysis modality affected the frequencies, but not the absolute numbers, of the B-cell subsets studied. These findings contradicted those of Ankersmit HJ et al. [24] but are consistent with those of Ducloux, D. et al. [25], who found modality-dependent immune populations [25]. The discrepancy was likely due to differences in sample composition and the relatively small sample size.

This study has a number of strengths and limitations for improvement.

It provided a comprehensive analysis of the phenotype of B cell-associated subsets in a cohort of dialysis patients with different durations of dialysis treatment.

In terms of limitations, it should be recognized that the pre-emptive control group was a small sample, as were the CAPD patients. The patient cohort came from a single transplant centre and consisted of a modest number of study participants. It is possible that a larger sample size would have yielded different statistical results. Regarding patient selection, the study included only kidney transplant candidates.

The results showed a significant decrease in the frequency of the naive and regulatory B cell subsets, tBregs, and plasmablasts in individuals with a longer history of dialysis. In contrast, the remaining B cell populations showed an increase, indicating a shift towards a memory B cell profile, which may be associated with susceptibility to infection [26,27]. In patients with a shorter history of dialysis, a profile of predominantly naive B cells was observed, accompanied by a high frequency of regulatory B cells, with a marked decrease in the memory profile. In absolute numbers, all B cell subsets showed an increasing trend in patients with a shorter duration of dialysis, whereas a significant decrease was observed in patients with a longer duration of dialysis.

The application of cluster analysis based on both frequencies and absolute numbers of B cell subsets allowed a comprehensive validation of the changes in the immune populations studied. In each case, distinct immune profiles were identified, demonstrating that analysis of immune cells in terms of both frequencies and absolute numbers provides more informative insights into the immune status of a heterogeneous cohort. Advances in B cell characterisation will improve our understanding of the role of B cells in transplant outcomes in these candidates, thereby facilitating the development of more effective treatment pathways.

The application of unsupervised machine learning clustering techniques to cluster design is a promising way to identify groups of patients with similar characteristics. This approach could facilitate investigations into whether the distribution of frequencies and absolute numbers of B-cell subset immunophenotypes in different clusters generated, in conjunction with age, dialysis duration, and dialysis modality, could potentially predict transplant outcome in the cohort studied during the follow-up of participants who received a kidney transplant.

In conclusion, cluster analysis could be used to classify kidney transplant candidates into distinct groups with unique immunophenotypic profiles. The architecture of these profiles varied depending on whether frequencies or absolute B-cell counts were considered. Therefore, it is recommended to consider both frequencies and absolute numbers of immunophenotypes when interpreting data. Our findings indicate that the distribution of B cell subsets is subject to variation according to the timing of dialysis initiation, the dialysis modality employed and the age of the patient. There is a clear association between changes in the frequencies and absolute numbers of B cell-associated subsets and dialysis vintage. It should be noted that frequencies are dependent on dialysis modality and patient age, whereas absolute numbers are independent of dialysis.

## Figures and Tables

**Table 1:** Clinical and demographical characteristics of the study sample. HD: Haemodialysis, CAPD: continuous ambulatory peritoneal dialysis.

**Patient characteristics**	n = 85
Male/Female, n (%)	58/27 (68.8/31.2%)
Age (years)	48.5 (19, 60)
**Primary kidney disease, n (%)**	
Hypertension	7 (8.2%)
Primary glomerulopathies	14 (16.5%)
Diabetes mellitus	8 (9.4%)
Urinary tract infections/stones	6 (7.1%)
Reflux nephropathy	10 (11.8%)
Polycystic kidney disease	15 (17.7%)
Other	12 (14%)
Unknown	13 (15.3%)
**Dialysis Data, n (%)**	
HD/CAPD	67/11 (86%/14%)
Dialysis vintage (months)	87 (34, 127)
Pre-emptive transplantation	7 (10.7%)
Retransplantation	2 (2.4%)

**Table 2:** Characteristics and variables of Model 1 (cluster 1 and cluster 2). In linear regression, *p* significant < 0.004. Ns = Non-significant.

Model 1 Variables of the Model	Cluster 1 n = 22 (28%) Median (IQR)	Cluster 2 n = 56 (72%) Median (IQR)	*p* Value Statistical Method
Naïve B cells, NBCs (% cells)	78 (69.85, 82.7)	56 (46.98, 64.88)	<0.001, linear regression
Total memory B cells, TMBCs (% cells)	13 (9.17, 16.52)	29 (20.2, 39.7)	<0.001, linear regression
Class-switched memory B cells, SMBCs (% cells)	9 (5.67, 11.5)	17 (11.15, 21.9)	<0.001, linear regression
Class non-switched memory B cells NSMBCs (% cells)	4 (2.8, 5.12)	11 (5.52, 15.45)	<0.001, linear regression
Peripheral equivalent to marginal zone B cells, MZBCs (% cells)	11 (4.47, 28.32)	33 (14.32, 40.1)	Ns, linear regression
Double negative with memory properties, DNMBCs (% cells)	8 (7, 11.78)	13 (9.6, 20.75)	Ns, linear regression
Plasmablasts, PL (% cells)	1 (0, 2.55)	0 (0, 0.5)	<0.001, linear regression
Transitional Bregs, tBregs (% cells)	3.4 (1.72, 10.65)	1 (0.2, 2.72)	=0.001, linear regression
Memory Bregs, mBregs (% cells)	4 (2.02, 5.05)	2 (0.1, 4.35)	Ns, linear regression
Dialysis vintage (months)	27.5 (12.5, 61.5)	115.5 (84.5, 130)	<0.001, linear regression
Age (years)	40.5 (25, 50)	54 (43, 58)	<0.001, linear regression
Type of dialysis (n, %)			0.03, Fisher’s test
HD	15 (68%)	51(91%)	
CAPD	7 (32%)	5 (9%)	
Creatinine (mg/dL)	5.1 (3.3, 6.8)	4.9 (4, 6.9)	Ns, Kruskal–Wallis

**Table 3:** Immune profile of the corresponding absolute numbers to frequencies of B cell subsets belonging to the two clusters of statistical model 1. Linear regression was used to compare the clusters, with *p* significant *p* < 0.005. Ns = Non-significant.

Variables of Model 1 Absolute Numbers of the Cells in Clusters of Model 1 Were Used (Cells/μL)	Cluster 1 n = 22 Median (IQR)	Cluster 2 n = 56 Median (IQR)	*p* Value *p* Significant < 0.005
Naïve B cells, NBCs (cells)	82 (35.56, 164.34)	51 (26.58, 82.27)	Ns, linear regression
Total memory B cells, TMBCs (cells)	14 (9.32, 21.71)	23 (15.21, 34.73)	0.004, linear regression
Class-switched memory B cells, SMBCs (cells)	8 (4.33, 14.96)	13 (9.77, 21.74)	Ns, linear regression
Class non-switched memory B cells, NSMBCs (cells)	5 (2.34, 7.96)	10 (5.38, 14.68)	0.004, linear regression
Double negative with memory properties, DNMBCs (cells)	10 (4.27, 17.24)	11 (8.14, 19.23)	Ns, linear regression
Peripheral equivalent to marginal zone B cells, MZBCs (cells)	2 (0.61, 2.72)	8 (2.9, 12.74)	0.001, linear regression
Plasmablasts, PL (cells)	0.09 (0, 0.32)	0 (0, 0.13)	Ns, linear regression
Transitional Bregs, tBregs (cells)	5 (1.66, 8.34)	1 (0.14, 2.89)	0.001, linear regression
Memory Bregs, mBregs (cells)	4 (1.81, 6)	2 (0.13, 4.4)	Ns, linear regression

**Table 4:** Significance difference of plasmablasts between dialysis patients and pre-emptives as seen in the linear regression model used for comparison. Reference category of the independent variable is the dialysis patients while the category compared is pre-emptive patients.

Dependent Variable	Independent Variable	Estimate	*p* Value	95% CI	Adjusted R Squared (R^2^)
Plasmablasts (percentages)	Intercept Pre-emptive	9.922 −4.608	0.0025 0.0017	(6.82, 13.02) (−6.208, −3.008)	0.275

**Table 5:** Variables included in statistical Model 2 (cluster A, cluster C), as well as the methodology employed for their comparative analysis. In linear regression, *p* < 0.004 was considered statistically significant. NS = Non-significant.

Model 2 Variables of the Model Cells/μL	Cluster A n = 32 (41%) Median (IQR)	Cluster C n = 46 (59%) Median (IQR)	*p* Value Statistical Method
Naïve B cells, NBCs (cells)	92 (49.13, 151.62)	42 (25.54, 70.26)	<0.001, linear regression
Total memory B cells, TMBCs (cells)	34 (23.84, 46.9)	15 (10.6, 22.6)	<0.001, linear regression
Class-switched memory B cells, SMBCs (cells)	19 (12.8, 26.38)	10 (6.69, 13.27)	=0.001, linear regression
Class non-switched memory B cells, NSMBCs (cells)	13 (8.36, 20.53)	6 (3.89, 7.96)	<0.001, linear regression
Peripheral equivalent to marginal zone B cells, peripheral equivalent to MZBCs (cells)	11 (2.72, 15.21)	3 (0.92, 7.67)	<0.001, linear regression
Double negative with memory properties cells, DNMBCs (cells)	17 (10.94, 25.22)	8 (4.47, 11.83)	0.003, linear regression
Plasmablasts (cells)	0.06 (0, 0.35)	0 (0, 0.1)	Ns, linear regression
Transitional Bregs (cells)	4 (1.15, 8.81)	1 (0.02, 2.21)	=0.001, linear regression
Memory Bregs (cells)	6 (2, 8.08)	1 (0.1, 2.49)	<0.001, linear regression
B lymphocytes (cells)	153 (103.53, 221.86)	65 (46.49, 102.99)	<0.001, linear regression
Months of dialysis (months)	62.5 (24,109.75)	112.5 (72.25, 129.75)	0.002, linear regression
Age (years)	51 (38.75, 58)	52 (41, 57.75)	Ns, Kruskal–Wallis
Type of dialysis (n, %)			Ns, Fisher’s test
HD	27 (84%)	39 (85%)	
CAPD	5 (16%)	7 (15%)	
Creatinine (mg/dL)	5.1 (3.9, 7.1)	4.7 (3.9, 6.7)	Ns, Kruskal Wallis

## Data Availability

Upon request, the corresponding author can provide the data sets used and analyzed in this study.

## Supplementary Materials

The following supporting information can be downloaded at: https://www.mdpi.com/article/10.3390/jcm13216454/s1, Table S1: B cell subsets phenotype and the antibodies used to identify the subsets; Table S2: Linear regression models for all variables in clusters of Model 1 compared with pre-emptive patients. Cluster 1: cluster 1 of Model 1 and the reference variable; cluster 2: cluster 2 of Model 1; cluster 3: group of pre-emptive patients; *p* significant < 0.004 via Bonferroni correction; Table S3: Characteristics of pre-emptive patients, including their relationship with clusters formed based on frequencies (Model 1) and absolute numbers (Model 2), as well as the statistical method used for comparisons. Statistically significant cases are indicated in bold. Ns = Non-significant; Table S4: Linear regression models for all variables in clusters of Model 2 for comparison with pre-emptive patients. Cluster A: cluster A of Model 2 and reference category; cluster C: cluster C of Model 2; cluster 3: group of pre-emptive patients; *p* significant < 0.004 via Bonferroni correction.
