# Supplementary material for: Frequencies or Absolute Numbers? Cluster Analysis of Frequencies and Absolute Numbers of B-Cell Subsets in Dialysis Patients Who Are Candidates for Kidney Transplantation Reveals Different Profiles
Source: J Clin Med. 2024 Oct 28;13(21):6454. doi: 10.3390/jcm13216454 (PMC11547170; doi:10.3390/jcm13216454)
Supplement: Supplementary file 1 [file jcm-13-06454-s001.zip › jcm-3246217-supplementary.pdf]

Title:

Frequencies or absolute numbers? Cluster analysis of frequencies and absolute numbers of B-cell subsets in dialysis patients who are candidates for kidney transplantation reveals different profiles.

Supplementary material

Cluster analysis:

Optimal variables for each model were determined after running the algorithm with different combinations of both continuous and categorical variables. Gower's distance and Ward's method were selected as the distance metric and linkage criterion, respectively, as this combination demonstrated optimal performance in the model and could also handle variables of mixed type (numeric and categorical). The results of the aforementioned analysis was used to group the transplant candidates.

We performed a comparative analysis of the clusters, coupled with an evaluation of the pre-emptive patients in the regression models. To do this, we used all of the variables in the model as individual dependent variables, including values for all patients, both those on dialysis and those not on dialysis (pre-emptive). The main independent variable was the clusters, consisting of the two clusters from each model, plus a third group (cluster of pre-emptives), representing the pre-emptive patients not included in the clustering model. In most models, all three clusters were used as predictor variables, except in the dialysis vintage model, where pre-emptive patients were excluded by definition. Possible confounding variables were also considered as independent variables for the construction of the final models through the process of purposive variable selection. Variables with a p-value less than 0.2 were included in the final model for each combination of independent variable and outcome. The Bonferroni correction was used for multiple comparisons.

Table S1: B cell subsets phenotype and the antibodies used to identify the subsets

B cell subset phenotypes	B cell population	Antibodies (clones) used for B cell subset phenotyping	Manufacturer
CD19+	total B lymphocytes	anti-CD19 PC5.5 clone J3-119.	Beckman Coulter Inc, Sykesville,MD, Usa
CD19+CD27+IgD− /+	Total memory B cells (TMBCs)	anti-CD27 PE-Dylight 594 clone LT27	EXBIO, Praha SA Praha SA; Czech Republic
CD19+CD27+IgM+IgD+	peripheral equivalent to marginal zone B cells, (MZBCs)	anti-IgD clone IA6-2	Thermo Scientific LSG Lagoas Park, Porto Salvo, Portugal
CD19+CD27+ IgD−	class-switched memory B cells (SMBCs)	anti-CD38-PB clone LS198-4-3	Beckman Coulter Beckman Coulter Inc., Sykesville, MD, USA
CD19+CD27+ IgD+	class-non switched memory B cells (NSMBCs)	anti-CD24-APC-Cy7 clone SN3	EXBIO, Praha SA Praha SA; Czech Republic
CD19+ CD27− IgD−	double negative (DNMBCs)	anti-CD45-PC7 clone J33	Beckman Coulter Beckman Coulter Inc., Sykesville, MD, USA
CD19+ CD27− IgD+ CD27++CD38++IgD+/− CD19+CD24++CD38++	naïve B cells (NBC) plasmablasts (PL) transitional regulatory B cells (tBregs)	anti-IgM PE clone SA-DA4	Beckman Coulter, Beckman Coulter Inc., Sykesville, MD, USA
CD19+CD24++CD27+	memory regulatory B cells, mBregs		

Table S2: Linear regression models for all variables in clusters of Model 1 compared with pre-emptive patients. Cluster 1: cluster 1 of Model 1 and the reference variable; cluster 2: cluster 2 of Model 1; cluster 3: group of pre-emptive patients; p significant < 0.004 via Bonferroni correction.

**Model 1 (percentages model)**					
**Dependent variable**	**Independent variables**	**Estimate**	**p value p significant <0.004**	**95% CI**	**Adjusted R-Squared (Adjusted R^2)**
Naïve B cells, NBCs (% cells)	Intercept<br>cluster2<br>cluster3	75.205<br>-21.072<br>-12.333	0.000<br>0.000<br>0.085	(68.287, 82.122)<br>(-29.236, -12.908)<br>(-26.413, 1.747)	0.226
Total memory B cells, TMBCs (% cells)	Intercept<br>cluster2<br>cluster3	13.700<br>16.163<br>8.843	0.000<br>0.000<br>0.083	(8.768, 18.632)<br>(10.342, 21.983)<br>(-1.195, 18.881)	0.256
Class-switched memory B cells, SMBCs (% cells)	Intercept<br>cluster2<br>cluster3	9.086<br>9.146<br>6.556	0.000<br>0.000<br>0.115	(5.064, 13.108)<br>(4.399, 13.893)<br>(-1.63, 14.743)	0.131
Class non-switched memory B cells, NSMBCs (% cells)	Intercept<br>cluster2<br>cluster3	4.609<br>7.221<br>2.291	0.002<br>0.000<br>0.444	(1.698, 7.52)<br>(3.786, 10.656)<br>(-3.633, 8.215)	0.165
Peripheral equivalent to marginal zone B cells, MZBCs (% cells)	Intercept<br>cluster2<br>cluster3	18.495<br>12.488<br>-9.610	0.000<br>0.012<br>0.256	(10.285, 26.706)<br>(2.799, 22.178)<br>(-26.321, 7.102)	0.111
Double negative with memory properties, DNMBCs, (% cells)	Intercept<br>cluster2<br>cluster3	10.495<br>5.442<br>4.305	0.000<br>0.018<br>0.270	(6.703, 14.288)<br>(0.967, 9.917)<br>(-3.414, 12.023)	0.044
Plasmablasts, PL (% cells)	Intercept<br>cluster2<br>cluster3	1.659<br>-1.327<br>1.055	0.000<br>0.000<br>0.059	(1.12, 2.198)<br>(-1.963, -0.691)<br>(-0.041, 2.152)	0.271
Transitional Bregs, tBregs (% cells)	Intercept<br>cluster2<br>cluster3	6.450<br>-4.307<br>5.093	0.000<br>0.000<br>0.014	(4.47, 8.43)<br>(-6.644, -1.970)<br>(1.062, 9.123)	0.271
Memory Bregs, mBregs (% cells)	Intercept<br>cluster2<br>cluster3	4.391<br>-0.121<br>6.295	0.002<br>0.941<br>0.028	(1.632, 7.149)<br>(-3.377, 3.134)<br>(0.680, 11.909)	0.048
Age (years)	Intercept<br>cluster2<br>cluster3	38.409<br>11.644<br>-0.838	0.000<br>0.000<br>0.871	(33.386, 43.433)<br>(5.716, 17.573)<br>(-11.063, 9.387)	0.169
Dialysis vintage (months)	Intercept<br>cluster2	-30.476<br>69.657	0.130<br>0.000	(-70.110, 9.159)<br>(47.340, 91.975)	0.328

Table S3: Characteristics of pre-emptive patients, including their relationship with clusters formed based on frequencies (Model 1) and absolute numbers (Model 2), as well as the statistical method used for comparisons. Statistically significant cases are indicated in bold. Ns= Non-significant.

**Pre-emptive patients (percentages) n = 7**	**Median (IQR)**	**Difference with clusters**	**Pre-emptive patients (absolute) n = 7**	**Median (IQR)**	**Difference with clusters**
Naïve B cells, NBCs (% cells)	68 (49.65,83.1)	Ns, linear regression	Naïve B cells, NBCs (cells)	28 (21.35, 117.79)	Ns, linear regression
Total memory B cells, TMBCs (% cells)	23 (9.85,28.45)	Ns, linear regression	Total memory B cells, TMBCs (cells)	16 (12.43, 27.28)	Ns, linear regression
Class-switched memory B cells, SMBCs (% cells)	9 (8.3,17.25)	Ns, linear regression	Class-switched memory B cells, SMBCs (cells)	10 (7.57, 16.63)	Ns, linear regression
Class non-switched memory B cells, NSMBCs (% cells)	4 (2.75,10.25)	Ns, linear regression	Class non-switched memory B cells, NSMBCs (cells)	6 (4.91, 10.64)	Ns, linear regression
Peripheral equivalent to marginal zone B cells, MZBCs (% cells)	9 (6.85,10.15)	Ns, linear regression	Peripheral equivalent to marginal zone B cells, MZBCs (cells)	5 (5, 5)	Ns, linear regression
Double negative with memory properties cells, DNMBCs (% cells)	8 (6.85,23.3)	Ns, linear regression	Double negative with memory properties cells, DNMBCs (cells)	8 (6.29, 15.23)	Ns, linear regression
Plasmablasts (% cells)	**1 (1,5.15)**	**Cluster 2, P < 0.001, linear regression**	Plasmablasts (cells)	**0.5 (0.24, 0.95)**	**Cluster A and C, p < 0.001, linear regression**

Transitional Bregs (% cells)	**11 (6.1,17.25)**	**Cluster 2, p < 0.001, linear regression**	Transitional Bregs (cells)	1 (0.36, 1.48)	Ns, linear regression
Memory Bregs (% cells)	7 (4.75,11.6)	Ns, linear regression	Memory Bregs (cells)	**0.3 (0.18, 0.81)**	**Cluster A, P < 0.001, linear regression**
Age (years)	38 (23.5,49.5)	Ns, linear regression	B lymphocyte s (cells)	106 (41.13, 155.18)	Ns, linear regression
Creatinine (mg/dl)	4.3 (3.5, 6)	Ns, Kruskal–Wallis	Age (years)	38 (23.5, 49.5)	Ns, Kruskal–Wallis
			Creatinine (mg/dl)	4.3 (3.5, 6)	Ns, Kruskal–Wallis

Table S4: Linear regression models for all variables in clusters of Model 2 for comparison with pre-emptive patients. Cluster A: cluster A of Model 2 and reference category; cluster C: cluster C of Model 2; cluster 3: group of pre-emptive patients; p significant < 0.004 via Bonferroni correction.

**Model 2 (absolute model)**					
**Dependent variable**	**Independe nt variables**	**Estimate**	**p value**	**95% CI**	**Adjusted R-Squared (Adjusted R^2)**
Naïve B cells, NBCs (cells)	Intercept<br>cluster A<br>cluster 3	48.582<br>64.637<br>19.899	0.000<br>0.000<br>0.432	(30.354, 66.811)<br>(36.178, 93.096)<br>(-30.259, 70.056)	0.180
Total memory B cells, TMBCs (cells)	Intercept<br>cluster A<br>cluster 3	16.233<br>21.562<br>9.042	0.000<br>0.000<br>0.139	(11.862, 20.605)<br>(14.737, 28.387)<br>(-2.987, 21.070)	0.309
Class-switched memory B cells, SMBCs (cells)	Intercept<br>cluster A<br>cluster 3	10.341<br>12.778<br>6.749	0.000<br>0.000<br>0.202	(6.550, 14.131)<br>(6.859, 18.696)<br>(-3.682, 17.180)	0.164
Class non-switched memory B cells, NSMBCs (cells)	Intercept<br>cluster A<br>cluster 3	5.923<br>8.897<br>2.320	0.000<br>0.000<br>0.412	(3.89, 7.957)<br>(5.722, 12.071)<br>(-3.275, 7.915)	0.259
Peripheral equivalent to marginal zone B cells MZBCs, (cells)	Intercept<br>cluster A<br>cluster 3	4.506<br>6.541<br>0.494	0.000<br>0.000<br>0.848	(2.652, 6.359)<br>(3.647, 9.434)<br>(-4.604, 5.593)	0.184
Double negative with memory properties, DNMBCs (cells)	Intercept<br>cluster A<br>cluster 3	10.705<br>14.339<br>1.246	0.000<br>0.001<br>0.868	(5.319, 16.092)<br>(5.929, 22.749)<br>(-13.575, 16.068)	0.106

Plasmablasts, PL (cells)	Intercept	0.082	0.655	(-0.282, 0.446)	0.156
	cluster A	0.156	0.587	(-0.412, 0.724)	
	cluster 3	2.097	0.000	(1.096, 3.098)	
Transitional Bregs, tBregs (cells)	Intercept	1.424	0.195	(-0.745, 3.593)	0.133
	cluster A	6.367	0.000	(2.981, 9.754)	
	cluster 3	-0.103	0.973	(-6.071, 5.866)	
Memory Bregs, mBregs (cells)	Intercept	1.560	0.003	(0.545, 2.575)	0.331
	cluster A	4.949	0.000	(3.364, 6.533)	
	cluster 3	-1.031	0.465	(-3.824, 1.761)	
B lymphocytes (cells)	Intercept	75.490	0.000	(53.352, 97.628)	0.283
	cluster A	102.884	0.000	(68.320, 137.447)	
	cluster 3	30.121	0.328	(-30.795, 91.037)	
Dialysis vintage (months)	Intercept	103.152	0.000	(87.882, 118.423)	0.084
	cluster A	-34.027	0.002	(-10.186, -2.843)	
